# Comparison of *Serenoa repens*, lycopene, and selenium versus dutasteride for the treatment of LUTS/BPH: an Italian multicenter case-control prospective study (COMP study)

**DOI:** 10.3389/fruro.2025.1565240

**Published:** 2025-04-25

**Authors:** Giuseppe Morgia, Arturo Lo Giudice, Sebastiano Cimino, Giulio Reale, Gaetano Larganà, Enza La Manna, Massimo Madonia, Alessandro Tedde, Francesco Santaniello, Giuseppe Vespasiani, Stefano Zaganelli, Salvatore Arnone, Nicola Cruciano, Maurizio Carrino, Francesco Persico, Carlo Terrone, Rafaela Malinaric, Andrea Minervini, Marco Carini, Giorgio Ivan Russo

**Affiliations:** ^1^ Urology Section, Department of Surgery, University of Catania, Catania, Italy; ^2^ ”S. Maria delle Croci” Hospital, Ravenna, Italy; ^3^ Urology Section, Department of Medicine, Surgery and Pharmacy, University of Sassari, Sassari, Italy; ^4^ Urology Section, “Ospedale Civile Fabriano” Hospital, Ancona, Italy; ^5^ Urology Section, University of Roma “Tor Vergata”, Rome, Italy; ^6^ Department of Urology, Lugo of Romagna Hospital, Ravenna, Italy; ^7^ Urology Section, “Maria Vittoria” Hospital, Torino, Italy; ^8^ Urology Section, “A. Cardarelli” Hospital, Napoli, Italy; ^9^ Dipartimento di Scienze Chirurgiche e diagnostiche integrate, Università degli studi di Genova, Genova, Italy; ^10^ IRCCS Ospedale Policlinico San Martino, Genova, Italy; ^11^ Urology Section, Department of Experimental and Clinical Medicine, University of Florence, Florence, Italy

**Keywords:** BPH, *Serenoa repens*, luts, prostate, 5ARI

## Abstract

**Introduction:**

Historically, medical therapy of BPH has had its foundations in alpha-blockers in monotherapy or in combination with 5 alpha reductase inhibitors (5-ARIs); in particular, two important trials (COMBAT and MTOPS) have demonstrated the pivotal role of combination therapy instead of monotherapy and have individuated which patients are most likely to benefit from alpha-blockers, 5-ARIs, or their combination. However different side effects of these drugs, such erectile dysfunction, ejaculatory disorders, loss of libido, could affect the adherence to treatment. In fact, SeR-Se-Ly would work by blocking 5-alpha reductase and the binding between the dihydrotestosterone and the androgen receptor, antagonizing the a1-adrenergic receptor, and preventing cell proliferation and the production of COX-2 and 5-leukotrienes. Prior randomized controlled trials confirmed the use of alpha-blockers in conjunction with Lipidic Extract (LE) of Serenoa Repens (SeR), specifically compounds containing both Selenium (Se) and Lycopene (Ly). Based on these premises, the aim of this Italian multicenter case-control prospective non-randomized study is to compare the therapy with SeR-Se-Ly alone versus Dutasteride alone against BPH, in order to research a good therapeutic alternative with limited side effects.

**Materials and methods:**

From April 2021 to April 2022, 269 consecutive patients from 21 Italian centers were enrolled in this case-control study. The inclusion criteria were: age between 55 and 80 years old, digital rectal examination negative for prostate nodules, PSA<<4 ng/ml, IPSS<>12, prostate volume >40 cc (assessed by ultrasound), Qmax <<15 ml/sec. Patients with prostate cancer, prior bladder cancer, diabetes mellitus, neurogenic disorders, severe liver disease, history of orthostatic hypotension or syncope, recent a-blocker treatment (within 1 month) or phytotherapy, including saw palmetto extract (within 3 months), prior medical therapy with 5-ARI or surgical treatment for LUTS/BPH, patients with catheter or QoL, Qmax measured at an episode of acute urine retention within the last 4 weeks, and patients with any ejaculatory disorders were excluded. During treatment period, patients received SeR-Se-Ly for one year (Group A), or Dutasteride 0.5mg for one year (Group B). The main outcome measures included International Prostatic Symptoms Score (IPSS) and IPSS quality-of -life (QoL), International Index of Erectile Function (IIEF-5), Qmax measured at uroflowmetry and the Male Sexual Health Questionnaire (MSHQ).

**Results:**

We have observed increasing of Qmax of 2 points in group A and 2,5 points group B; IPSS has been reduced of 4 points in group A and 4,5 points in group B; IIEF-5 didn’t change in group A and it has been reduced in group B by -1; regarding the MSHQ, only the item MSHQ-Satisfaction has changed in group A: -1, in group B MSHQ was reduced by -2 points in ED, by -5 points in EJ, by -2,5 points in Satisfaction, by -0,5 points in Intercourse, by -3,2 points in Desire.

**Conclusions:**

SeR-Se-Ly could be a valid alternative to dutasteride, providing comparable effects on symptoms related to LUTS/BPH and avoiding sexual dysfunctions. SeR-Se-Ly should not be substituted for dutasteride in those patients for whom prostate volume reduction is desired.

## Introduction

About 50% of men between 50 and 60 years old are affected by Lower Urinary Tract Symptoms (LUTS) due to benign prostatic hyperplasia (BPH), this percentage increase linearly with age (1). BPH is the results of the summary of different and complex mechanisms, involving hormonal pathways but also inflammation pathways: testosterone, produced by the testes, is converted into dihydrotestosterone (DHT) by the enzyme 5-alpha reductase within the prostate gland. DHT binds to androgen receptors in the prostate cells, leading to cellular proliferation and growth; estrogen, primarily derived from the conversion of testosterone by the enzyme aromatase, also contributes to the growth of the prostate gland. Estrogen receptors are present in prostatic tissue and can promote cell proliferation, increase smooth muscle tone, and stimulate the production of growth factors; Insulin-like growth factor (IGF) particularly IGF-1, are growth factors that play a role in cell proliferation, differentiation, and survival. These growth factors are produced within the prostate tissue and are regulated by androgen signaling. IGFs can stimulate prostate cell growth and contribute to the development of BPH; transforming growth factor-beta (TGF-β) is a multifunctional cytokine that regulates cell growth, differentiation, and apoptosis. Alterations in the TGF-β pathway have been associated with BPH development; chronic inflammation is believed to play a role in BPH development and progression. Inflammatory cytokines, such as interleukin-6 (IL-6), tumor necrosis factor-alpha (TNF-α), and prostaglandins, can affect the hormonal balance in the prostate gland and contribute to tissue remodeling and hyperplasia, the inflammatory cells in fact, produce growth factors such as VEGF or TGF- b, which can support the fibromuscular growth in BPH (2, 3). Historically, medical therapy of BPH has had its foundations in alpha-blockers in monotherapy or in combination with 5 alpha reductase inhibitors (5-ARIs); in particular, two important trials (COMBAT and MTOPS) have demonstrated the pivotal role of combination therapy instead of monotherapy and have individuated which patients are most likely to benefit from alpha-blockers, 5-ARIs, or their combination (4, 5). However different side effects of these drugs, such erectile dysfunction, ejaculatory disorders, loss of libido, could affect the adherence to treatment, despite the benefits on symptoms; indeed, minimally invasive surgical therapies (MIST) are constantly being sought that can reduce or eliminate any side effects and that can be followed in ambulatorial settings (6).

While 5-ARIs primarily function by inhibiting 5-alpha reductase to reduce prostate volume, SeR-Se-Ly has a broader mechanism of action, including inhibition of 5-alpha reductase, blocking of DHT binding to androgen receptors, alpha-adrenergic receptor antagonism, anti-inflammatory effects, and inhibition of COX-2 and leukotrienes. These combined actions contribute to symptom relief while avoiding the sexual dysfunction side effects commonly seen with 5-ARIs.

Previous randomized controlled trial validated the use of alpha-blockers in combination with Lipidic Extract (LE) of Serenoa Repens (SeR) and, in particular, compounds containing also Selenium (Se) and Lycopene (Ly) against BPH symptoms (7); indeed, SeR-Se-Ly would act by inhibiting the 5-alpha reductase and the binding between the dihydrotestosterone and the androgen receptor, antagonizing the a1-adrenergic receptor, and inhibiting cell proliferation and the production of COX-2 and 5-leukotrienes (8).

Based on these premises, the aim of this italian multicenter case-control prospective non-randomized study is to compare the therapy with SeR-Se-Ly alone versus Dutasteride alone against BPH, in order to research a good therapeutic alternative with limited side effects.

## Materials and methods

### Study design

From April 2021 to April 2022, 269 consecutive patients from 21 Italian centers were enrolled in this case-control study. The following study was conducted in accordance with the ethical principles described in the Declaration of Helsinki.

### Participants

The inclusion criteria were: age between 55 and 80 years old, digital rectal examination negative for prostate nodules, PSA<=4 ng/ml, IPSS>12, prostate volume >40 cc (assessed by ultrasound), Qmax <<15 ml/sec. Patients with prostate cancer, prior bladder cancer, diabetes mellitus, neurogenic disorders, severe liver disease, history of orthostatic hypotension or syncope, recent a-blocker treatment (within 1 month) or phytotherapy, including saw palmetto extract (within 3 months), prior medical therapy with 5-ARI or surgical treatment for LUTS/BPH, patients with catheter or QoL, Qmax measured at an episode of acute urine retention within the last 4 weeks, and patients with any ejaculatory disorders were excluded. After screening and possible pharmacological wash-out, the participants were divided in two arms consisting in 124 patients for SeR-Se-Ly therapy and 145 patients for dutasteride therapy.

### Intervention

During treatment period, patients received SeR-Se-Ly for one year (Group A), or Dutasteride 0.5mg for one year (Group B).

### Clarification on group assignment

The treatment choice was based on shared decision-making between the physician and patient, considering individual preferences and clinical profiles.

### Outcomes

**Figure 1:**
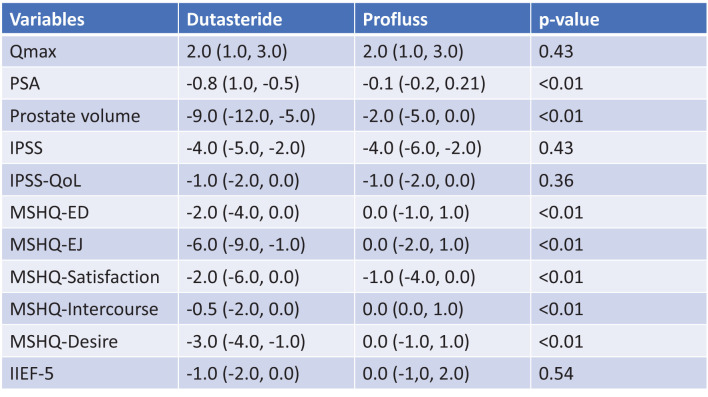
Median changes of primary and secondary endpoints from baseline to 1 year.

The main outcome measures included International Prostatic Symptoms Score (IPSS) and IPSS quality-of -life (QoL) (9), International Index of Erectile Function (IIEF-5) (10), Qmax measured at uroflowmetry and the Male Sexual Health Questionnaire (MSHQ) (11), performed at enrollment (Baseline), 3 month (visit one), at 6 months (visit two), 9 moths (visit three), 12 months (visit four). At baseline, visit two, visit three, and visit four, prostate specific antigen (PSA) tests and ultrasonography were used to assess the prostate’s volume. A bladder full of 150 and 550 ml and a voided volume of 125 ml were necessary for the uroflowmetry to be performed with a meaningful measurement of Qmax. Adverse events (AEs) were collected in order to evaluate safety data. Adverse events related to treatment (TEAEs) were defined as those that were reported for the first time or that got worse after beginning therapy. The primary endpoints of the study were the clinically significant reduction of IPSS, clinically significant increase of Qmax and the change in erectile function (assessed by IIEF5 and MSHQ) in patients treated with SeR-Se-Ly compared to Dutasteride group after 1 year. Secondary endpoints of the study were considered prostate volume, and serum PSA. One tablet of SeR-Se-Ly consisted of 320mg of supercritical CO2 lipidic extract SeR containing 85% of fatty acids sterols, selenium (50 mcg) and lycopene (5 mg). The study design is resumed in **Figure 1**.

### Statistical analysis

The sample size was estimated based on the previous study (7). A normality test was conducted before choosing non-parametric methods; specifically, the Shapiro-Wilk test was applied to assess data distribution. Given that some of our variables were not normally distributed, non-parametric methods (Mann-Whitney U Test and Chi-Square Test) were deemed appropriate. Changes from visit one to visit two, from two to four relatives to primary endpoints, and from baseline to visit four relatives to primary and secondary endpoints were used to test the efficacy variables.

The efficacy variables were tested by analyzing changes from visit one to visit two, and from baseline to visit four, using non-parametric tests where appropriate.

The rank analysis of covariance was used to adjust for baseline differences between groups.

The Chi-Square Test (x2-Test) was used to test qualitative results, while the z-test was used to compare two proportions. For all tests, a two-sided P-value <0.05 was deemed statistically significant. The impact of the difference value at the baseline was assessed using the rank analysis of covariance.

## Results

All of patients have completed the follow-up of 12 months. The baseline characteristics of groups, including IIEF-5 and MSHQ are summarized in **Table 1**; of all subjects, average age was 65 years (range: 57–72), average PSA was 2.3 ng/ml (range: 1.9–3.2), average Qmax was 10.9 (range: 9–13), average prostate volume was 53 cc (range: 42–68), average IPSS was 19 (range: 13–21) (**Figure 1**).

**Table 1:** Baseline characteristics of groups.

Variables	Group A SeR-Se-Ly	Group B Dutasteride	p-value
Age	63.0 (57.0-69.0)	66 (63.0-72.0)	<0.01
Qmax	12.0 (10.0-13.0)	10.0 (9.0-12.0)	<0.01
PSA	1.93 (1.22-2.85)	2.7 (1.9-3.2)	<0.01
Prostate volume	49.0 (42.0-55.0)	57.0 (50.0-68.0)	<0.01
IPSS	18.0 (13.0-19.0)	19.0 (16.0-21.0)	<0.01
IPSS-QoL	3.0 (2.0-4.0)	4.0 (3.0-5.0)	<0.01
MSHQ-ED	16.0 (14.0-18.0)	14.0 (12.0-16.0)	<0.01
MSHQ-EJ	34.0 (30.0-38.0)	33 (29.0-36.0)	<0.01
MSHQ-Satisfaction	24.0 (20.0-24.0)	22 (18.0-24.0)	<0.01
MSHQ-Intercourse	8.0 (7.0-8.0)	8.0 (7.0-8.0)	0.14
MSHQ-Desire	16.0 (13.0-16.0)	15.0 (13.0-16.0)	<0.01
IIEF-5	19.0 (15.0-21.0)	15.0 (12.0-20.0)	<0.01

**Figure 2:**
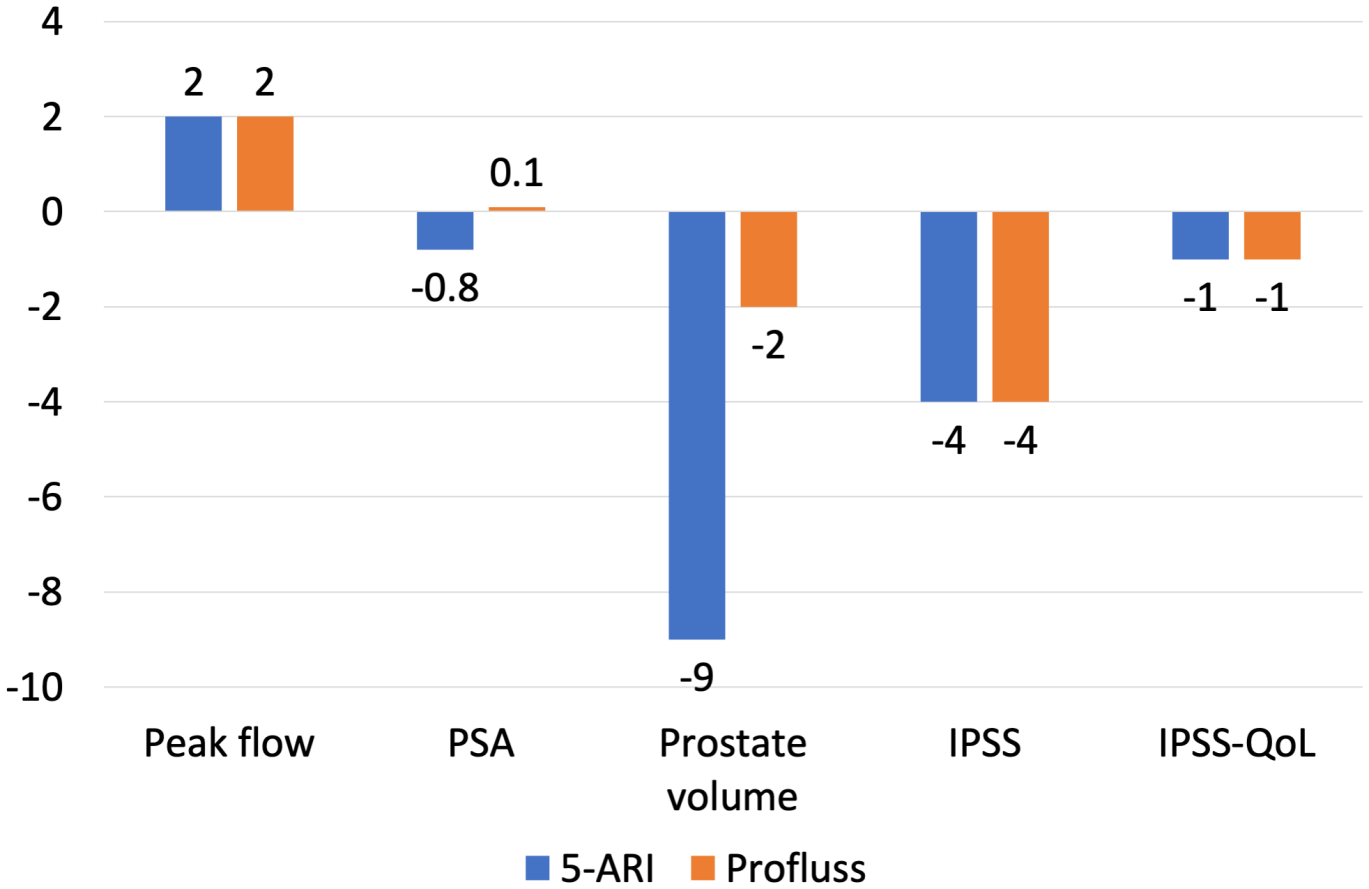
Changes of peak flow, prostate volume, IPSS, IPSS-QoL from baseline to 6-months.

At six months of follow-up, we have observed increasing of Qmax of 2 points (range 1;3) in group A and group B; IPSS has been reduced of 4 points in both groups (range 5;2 for group B and range 6;2 for group A). Results at six months are showed in **Figure 2**.

**Figure 3:**
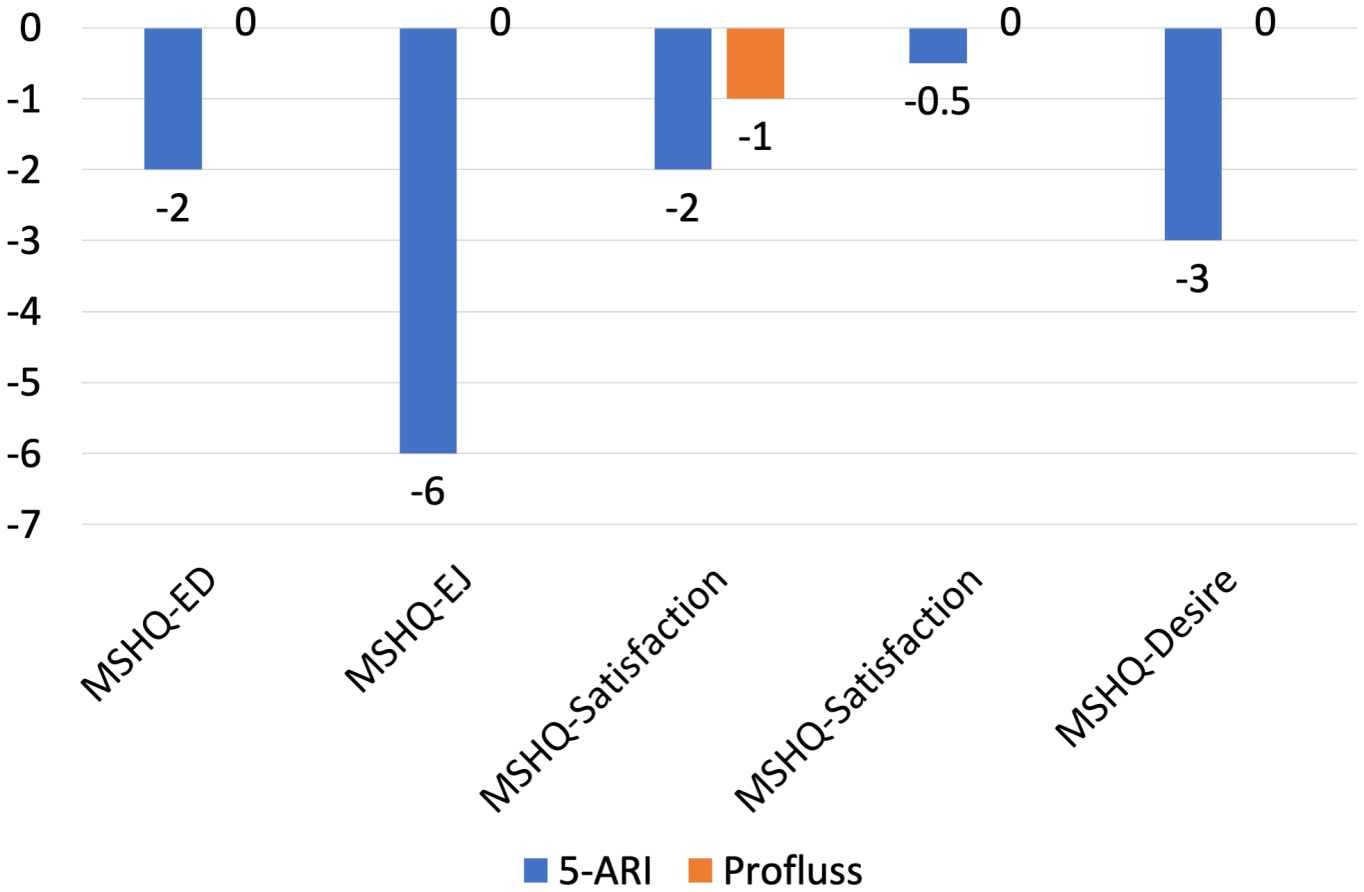
Changes of MSHQ sub-domains from baseline to 6 months.

IIEF-5 didn’t change in group A and it has been reduced in group B by -1 (-2;0); regarding the MSHQ, only the item MSHQ-Satisfaction has changed in group A: -1 (-4;0), in group B MSHQ was reduced by -2 (-4;0) points in erectile dysfunction (ED), by -6 (-9;-1) points in ejaculation (EJ), by -2 (-6;0) points in Satisfaction, by -0,5 (-2;0) points in Intercourse, by -3 (-4;-1) points in Desire. All these results are statistically significant due P value <0.01 (**Figure 3**).

**Figure 4:**
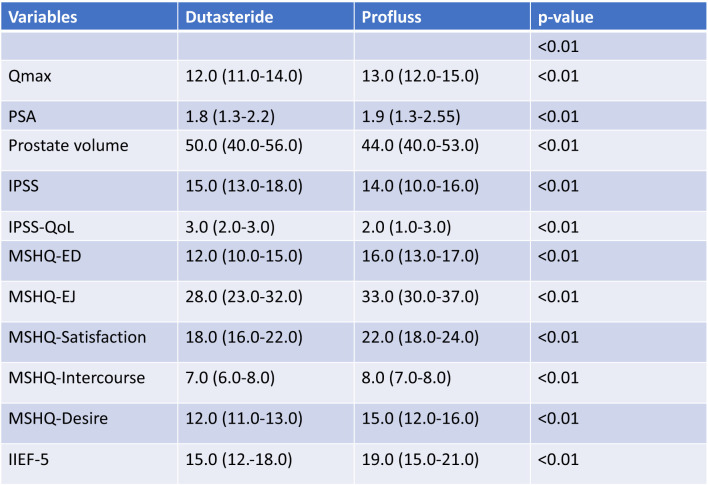
Median values after 1 year.

At one year of follow-up, we have observed increasing of Qmax of 2 points in group A and 2,5 points group B; IPSS has been reduced of 4 points in group A and 4,5 points in group B; IIEF-5 didn’t change in group A and it has been reduced in group B by -1; regarding the MSHQ, only the item MSHQ-Satisfaction has changed in group A: -1, in group B MSHQ was reduced by -2 points in ED, by -5 points in EJ, by -2,5 points in Satisfaction, by -0,5 points in Intercourse, by -3,2 points in Desire. All these results are statistically significant due P value <0.01. Results at one year are showed in **Figure 4**.

### Secondary endpoint

We observed significant differences between group A and group B in terms of variation of PSA and prostate volume: at six months we have observed PSA reduction of -0,1ng/ml in patients of group A and -0,8 ng/ml in patients of group B, prostate volume reduction of -2ml in patients of group A and -9ml in patients of group B (**Figure 2**); at twelve months we have observed PSA reduction of -0,1ng/ml in patients of group A and -1,2 ng/ml in patients of group B, prostate volume reduction of -2ml in patients of group A and -20ml in patients of group B (**Figure 3**). All these results are statistically significant due P value <0.01.

## Discussion

The mechanism of action of plant-based compounds on BPH are not fully understood, despite that several studies have related prostate health with diet and inflammation. Diet habits could help to prevent Prostate cancer (PCa) and BPH (12), the Mediterranean diet could provide various compounds such phytoestrogens, flavonoids, lycopene that could be useful for prevention of PCa (13, 14) and BPH (12, 15); inflammation is a key point in developing BPH (3) and PCa through activation of different pathways (16–19). PCa is a disease strongly influenced by genetics (20, 21), but BPH could be influenced by acting on inflammation, in fact some studies have demonstrated the potential therapeutic effect of SeR-Se-Ly on chronic prostatitis and pelvic pain syndrome related to BPH (10, 22).

Based on these evidences, European Association of Urology guidelines suggest proposing SeR compounds to men with LUTS who want to avoid any potential adverse events especially related to sexual function, but this recommendation is weak (1).

In our study, we observed that the therapeutic effect of SeR-Se-Ly is already present after six months of follow-up. In fact, a significant reduction in IPSS and an increase in Qmax was observed, this therapeutic effect was maintained even after one year of therapy. Comparing these therapeutic effects to those observed in the group treated with dutasteride, we observed that the two groups had comparable benefits from the two therapies administered. We focused our attention on the analysis of Dutasteride side effects concerning sexual function, analyzed by MSHQ; we observed that the most predominant side effect was worsening of ejaculatory function, followed by desire, erectile dysfunction and satisfaction. These side effects were already present at the six-month follow-up and slightly worsened after one year of therapy, while they were absent in the group of patients treated with SeR-Se-Ly. Our secondary outcomes were the reduction of PSA and prostate volume, from this point of view Dutasteride proved to be clearly superior to SeR-Se-Ly.

While our study suggests that SeR-Se-Ly provides comparable symptom relief to Dutasteride, we acknowledge that prostate volume reduction was significantly greater in the Dutasteride group. This aligns with prior studies suggesting that prostate volume reduction correlates with long-term symptom relief and reduced risk of acute urinary retention. However, our findings indicate that patients who prioritize symptom control without the adverse effects on sexual function may benefit from SeR-Se-Ly.

The therapeutic effect of SeR-Se-Ly, comparable to Dutasteride, is probably linked to the synergistic effect of the three compounds contained within it. One suggested mechanism involves the inhibition of the enzyme 5-alpha reductase. This enzyme converts testosterone into dihydrotestosterone (DHT), a hormone that promotes the growth of prostate tissue. By inhibiting 5-alpha reductase, SeR may help reduce DHT levels, leading to a decrease in prostate gland enlargement. In addition to its potential impact on 5-alpha reductase, SeR may also exert anti-inflammatory effects. Chronic inflammation is thought to contribute to the development and progression of BPH. SeR extract has been shown to inhibit the production of inflammatory markers such as interleukin-6 (IL-6) and tumor necrosis factor-alpha (TNF-alpha) in prostate cells, thereby reducing inflammation in the prostate gland (23–25). Selenium is an essential trace mineral that acts as a cofactor for several antioxidant enzymes, including glutathione peroxidase. The antioxidant properties of selenium help protect cells from oxidative damage caused by reactive oxygen species (ROS). In BPH, oxidative stress plays a role in the pathogenesis and progression of the condition. By reducing oxidative stress, selenium may help mitigate the inflammation and cellular damage associated with BPH. Furthermore, selenium has been implicated in modulating the expression of genes involved in cell proliferation and apoptosis. It may regulate the expression of genes associated with the cell cycle, such as cyclins and cyclin-dependent kinases (CDKs). This modulation of gene expression can potentially impact the growth and survival of prostate cells, thereby influencing the progression of BPH (26–28). Lycopene is a potent antioxidant that is particularly abundant in tomatoes. Its antioxidant properties enable it to scavenge free radicals and protect cells from oxidative damage. Oxidative stress in the prostate gland can contribute to the development and progression of BPH. By reducing oxidative stress, lycopene may help mitigate the cellular damage and inflammation associated with BPH. Moreover, lycopene has been shown to influence various molecular pathways involved in cell growth and proliferation. It can modulate the expression of genes associated with cell cycle regulation, such as cyclins, cyclin-dependent kinases, and tumor suppressor genes. These effects may help inhibit the growth of prostate cells and reduce the enlargement of the prostate gland (29, 30).

## Conclusions

SeR-Se-Lycould be a valid alternative to dutasteride, providing comparable effects on symptoms related to LUTS/BPH and avoiding sexual dysfunctions. SeR-Se-Ly should not be substituted for dutasteride in those patients for whom prostate volume reduction is desired. We believe that this study could be useful for directing urologists to obtain a higher patient compliance with pharmacological therapy. The main limitation of our study is that it is not a randomized clinical trial and that the drug administration was not double-blind. However, we believe that it can be a good starting point to explore the topic in question and that it can be a spur for the organization of randomized clinical trials.

## Data Availability

The original contributions presented in the study are included in the article/supplementary material, further inquiries can be directed to the corresponding author/s.
